# Anomalous diffusion of Ibuprofen in cyclodextrin nanosponge hydrogels: an HRMAS NMR study

**DOI:** 10.3762/bjoc.10.286

**Published:** 2014-11-19

**Authors:** Monica Ferro, Franca Castiglione, Carlo Punta, Lucio Melone, Walter Panzeri, Barbara Rossi, Francesco Trotta, Andrea Mele

**Affiliations:** 1 Department of Chemistry, Materials and Chemical Engineering “G. Natta”, Politecnico di Milano, Piazza L. da Vinci 32 – 20133 Milano Italy; 2 CNR-ICRM, Via L. Mancinelli, 7 20131 Milano, Italy; 3 Elettra - Sincrotrone Trieste, Strada Statale 14 km 163.5, Area Science Park, 34149 Trieste, Italy and Department of Physics, University of Trento, via Sommarive 14, 38123 Povo, Trento, Italy; 4 Department of Chemistry, University of Torino, Via Pietro Giuria 7, 10125 Torino, Italy

**Keywords:** cross-linked polymers, cyclodextrin nanosponges, diffusion, HRMAS NMR spectroscopy, TEM

## Abstract

Ibuprofen sodium salt (IP) was encapsulated in cyclodextrin nanosponges (CDNS) obtained by cross-linking of β-cyclodextrin with ethylenediaminetetraacetic acid dianhydride (EDTAn) in two different preparations: CDNSEDTA 1:4 and 1:8, where the 1:*n* notation indicates the CD to EDTAn molar ratio. The entrapment of IP was achieved by swelling the two polymers with a 0.27 M solution of IP in D_2_O, leading to colourless, homogeneous hydrogels loaded with IP. The molecular environment and the transport properties of IP in the hydrogels were studied by high resolution magic angle spinning (HRMAS) NMR spectroscopy. The mean square displacement (MSD) of IP in the gels was obtained by a pulsed field gradient spin echo (PGSE) NMR pulse sequence at different observation times *t*_d_. The MSD is proportional to the observation time elevated to a scaling factor α. The α values define the normal Gaussian random motion (α = 1), or the anomalous diffusion (α < 1, subdiffusion, α > 1 superdiffusion). The experimental data here reported point out that IP undergoes subdiffusive regime in CDNSEDTA 1:4, while a slightly superdiffusive behaviour is observed in CDNSEDTA 1:8. The transition between the two dynamic regimes is triggered by the polymer structure. CDNSEDTA 1:4 is characterized by a nanoporous structure able to induce confinement effects on IP, thus causing subdiffusive random motion. CDNSEDTA 1:8 is characterized not only by nanopores, but also by dangling EDTA groups ending with ionized COO^−^ groups. The negative potential provided by such groups to the polymer backbone is responsible for the acceleration effects on the IP anion thus leading to the superdiffusive behaviour observed. These results point out that HRMAS NMR spectroscopy is a powerful direct method for the assessment of the transport properties of a drug encapsulated in polymeric scaffolds. The diffusion properties of IP in CDNS can be modulated by suitable polymer synthesis; this finding opens the possibility to design suitable systems for drug delivery with predictable and desired drug release properties.

## Introduction

Cyclodextrin nanosponges (CDNS) are a novel, promising class of nanoporous, three-dimensional polymers with interesting properties of sorption of both organic and inorganic species [1–3]. Indeed, several examples of applications can be found in the recent literature, including biocatalysis [4], agriculture [5], environmental control [6] and pharmaceutical applications such as drug stabilization, enhancement of bioavailability and drug delivery [7–11]. A typical synthetic protocol for their synthesis consists of the condensation of OH groups of the glucose units of cyclodextrin (CD) with a suitable, poly-functional cross-linker agent, generally an activated derivative of a tetracarboxylic acid, such as ethylenediaminetetraacetic acid dianhydride (EDTAn) [12], pyromellitic anhydride (PMA), or a phosgene synthetic equivalent as carbonyldiimidazole (CDI) or diphenyl carbonate (DPC) [1–3]. For clarity, the corresponding nanosponge will be indicated as CDNS followed by the acronym of the cross-linker agent (e.g., CDNSEDTA for cyclodextrin nanosponge polymerized with the EDTA derivative). The growth of the polycondensation products leads to a statistic three-dimensional network characterized by different types of cavities, namely the apolar cavity of the CD units and the pores of the growing polymer. As previously stressed, CDNS are, in the majority of cases, completely – or almost completely – amorphous [13], thus preventing the possibility of structural assessment via diffraction methods and making a thorough structural characterization a still open investigation field. The main structural features have been obtained, so far, by a combined use of solid state ^13^C CP-MAS NMR, FTIR and Raman spectroscopies [14–16].

CDNS are per se not soluble, due to the extended three-dimensional covalent network. However, in many cases they showed interesting swelling properties when contacted with water or water solution only, affording hydrogels. The swelling ability of CDNS depends on different factors, such as the chemical compositions of the nanosponge (e.g., CDNSPMA, CDNSEDTA are swellable, CDNSDPC are not) and the cross-linker to CD mole ratio *n* [17]. Recently, we demonstrated that the CDNS hydrogels undergo phase transitions by changing the hydration level *h* = *m*(H_2_O)/*m*CDNS by a complex interplay of physical and chemical interactions [17–18]. The formation of hydrogel from CDNS is of particular practical importance as it is an easy and efficient way to load the gel with a given compound by swelling the polymer in an aqueous solution of the molecule of interest.

In the past few years the potential of CDNS as drug carriers has been adequately underlined and recently reviewed in this journal [19]. Indeed, CDNS can encapsulate either lipophilic or hydrophilic active pharmaceutical ingredients, protect them against undesired degradation, enhance water solubility when necessary, facilitate their gradual release over extended times, thus increasing the bioavailability at the target site [19]. In this scenario of growing importance of CDNS in pharmaceutical formulations as drug container and/or carrier, it is of paramount importance a clear understanding, at molecular level, of the state of the confined drug inside the polymeric network, especially in the gel state. To this end, the use of high resolution magic angle spinning (HRMAS) NMR spectroscopy [20] opened the possibility of using the whole repertoire of NMR experiments to spot on the structural and dynamic properties of the drug entrapped in the polymeric framework. HRMAS NMR spectroscopy has become an extremely versatile technique that provides high resolution NMR data on heterogeneous suspensions [21], gels [22] and swellable solids [23]. The basic principle is simple and can be summarized as follows: the dramatic line broadening due to dipolar relaxation and magnetic susceptibility inhomogeneity present in semi-solid or in heterogeneous samples can be conveniently averaged to small or null values by orienting the sample at the magic angle (β = 54.7°) with respect to static B_0_ field and by spinning the sample at a rate in the range of 2–10 kHz. These conditions are generally achieved by using commercially available HRMAS probe-heads in a high resolution NMR spectrometer. The sample is generally prepared in the usual solid state rotors. Under these conditions, NMR spectra with a typical resolution in the liquid state can be obtained from samples not suitable for routine liquid state NMR. Cutting-edge examples taken from the recent literature include metabolomics [24–25], structure of organic ligands bound to a solid support [26], and catalysis [27].

In addition to providing structural information, HRMAS NMR has also been used to investigate transport phenomena in heterogeneous systems endowed with liquid-like dynamics by applying pulsed field gradient spin echo (PGSE) methodologies under magic-angle spinning conditions [23]. This point is of great interest in the field of controlled release of active pharmaceutical ingredients (API), as one of the possible release mechanisms can be based on diffusion. The possibility of measuring the transport properties of the API by a direct method (NMR) and in the polymeric matrix effectively used for the formulation is therefore a physicochemical parameter directly related to the potential use in therapy.

In this work we present a study based on HRMAS NMR spectroscopy on the transport properties of Ibuprofen sodium salt (IP) confined in CDNSEDTA nanosponge hydrogels. Here, two different formulation of the nanosponges are investigated, characterized by a different CD/cross linker molar ratio: 1:4 (CDNSEDTA 1:4) and 1:8 (CDNSEDTA 1:8). The main purpose of the work is to spot on the dynamic properties of this popular analgesic, non-steroidal anti-inflammatory drug, in a potentially useful cross-linked scaffold for advanced formulations. The results point out that the diffusive regimes of IP in the hydrogels strongly depend on the polymeric network features and allow a modulation of the diffusivity as a function of the polymer formulation.

## Results and Discussion

### HRMAS NMR spectra of IP confined in CDNSEDTA

**Figure 1:**
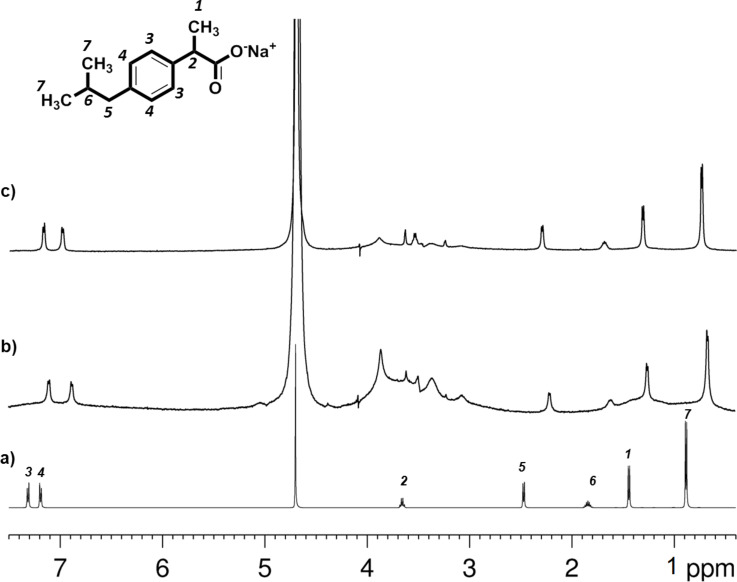
a) ^1^H high resolution NMR spectrum of IP dissolved in D_2_O, b) ^1^H HRMAS NMR spectrum of IP-CDNSEDTA (1:4) sample, c) ^1^H HRMAS NMR spectrum of IP-CDNSEDTA (1:8) sample.

The ^1^H HRMAS NMR spectra of IP in CDNSEDTA (1:4) and CDNSEDTA (1:8) polymer systems are shown in Figure 1 together with the ^1^H NMR spectrum of IP dissolved in D_2_O. The molecular formula of IP (sodium salt, racemic mixture) and the atom numbering are also shown.

Noticeably, the HRMAS spectra shows well resolved lines for the IP molecule, comparable with the high resolution spectrum, while CDNS gives broad overlapped resonances spanning the range 3–4 ppm due to the slow mobility of the polymer network. The chemical shifts (δ) of IP in D_2_O solution and in CDNS gel are reported in Table 1. Chemical shift variations (Δδ) of IP signals are observed on passing from the liquid D_2_O solution to the CDNS gel system, thus reflecting a different molecular environment experienced by the drug molecule and interactions with the polymeric backbone. Incidentally, no particular doubling of signals was observed due to the interaction of the racemic IP with the chiral cavity of CDs in the nanosponge gels. Conversely, significant doubling of selected aromatic and methyl protons of IP was reported to take place in the presence of monomeric β-CD in D_2_O solution, thus confirming, in that case, the formation of an inclusion complex and the chiral discrimination [28]. The absence of signal doubling in the case described in the present work can be due to either the absence of significant inclusion of IP in the CD cavity, or to unresolved splitting due to broader signals. The data of Table 1 show an upfield shift (lower δ value) in both gel networks, and this shift is more pronounced in CDNSEDTA (1:4). The chemical shift variations are more relevant for the aromatic H(4) Δδ = 0.2–0.3 ppm upfield in gel, for the H(3) Δδ = 0.14–0.2 and for the aliphatic H(6) Δδ = 0.17–0.25, thus locating in the aromatic part of IP the major site of interaction with the polymeric network.

**Table 1:** Chemical shift (δ) of IP dissolved in D_2_O solution, and confined in CDNSEDTA (1:4) and CDNSEDTA (1:8).

sample	chemical shifts (δ, ppm) and multiplicity of IP protons						
	3 (d)	4 (d)	2 (q)	5 (d)	6 (m)	1 (d)	7 (d)

D_2_O solution	7.31	7.19	3.66	2.47	1.87	1.45	0.89
CDNSEDTA 1:8	7.17	6.99	3.54	2.30	1.70	1.32	0.74
CDNSEDTA 1:4	7.11	6.88	n.d.	2.21	1.62	1.26	0.67

### HRMAS NMR diffusion measurements

Before any discussion on diffusivity of IP derived from NMR data, it is worth summarizing here some key points on the diffusion theory and the way diffusion can be studied by NMR. The basic pulsed field gradient spin-echo (PGSE) experiments allow to measure the molecular mean square displacement (MSD) along the axis of the pulsed gradient (usually *z*-axis) with chemical specificity. The PGSE sequence is based on a first defocussing gradient of duration δ (little delta, few milliseconds. This quantity is not to be confused with the chemical shift, indicated by the same symbol in the text), followed by a free precession delay during which diffusion takes place (big delta, Δ), and a final refocusing gradient δ to achieve the echo. The measurement is made over an overall observation time *t*_d_, (*t*_d_ = Δ−δ/3) and the molecular mean square displacement (MSD) along the *z* reference axis 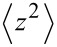 can be calculated by fitting the gradient dependent signal intensities *I(q,t*_d_*)* according to Equation 1,

[1]
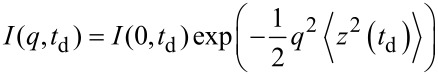


where q = γδg, γ is the magnetogiric ratio of the observed nucleus, g is the applied field gradient, and δ is the gradient pulse length. In the order, the first term is a constant, the second and third are user controlled instrumental parameters. The MSD of the diffusing molecule is proportional to the observation time *t*_d_:

[2]
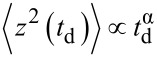


The diffusion processes may be grouped in different classes, depending on the value of the exponent α: i) isotropic unrestricted diffusion when α = 1, ii) anomalous subdiffusive regime for 0 < α < 1, iii) anomalous superdiffusive regime for α > 1. Recently, it was underlined the importance of anomalous diffusion behaviours in complex systems such as polymeric networks of supramolecular assemblies. Noticeable examples reported in the recent literature deal with Pluronic F127 gel [29], microemulsions [30], and layered media [31].

Equation 1 can be easily transformed in Equation 3:

[3]
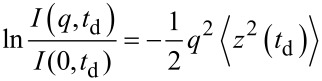


**Figure 2:**
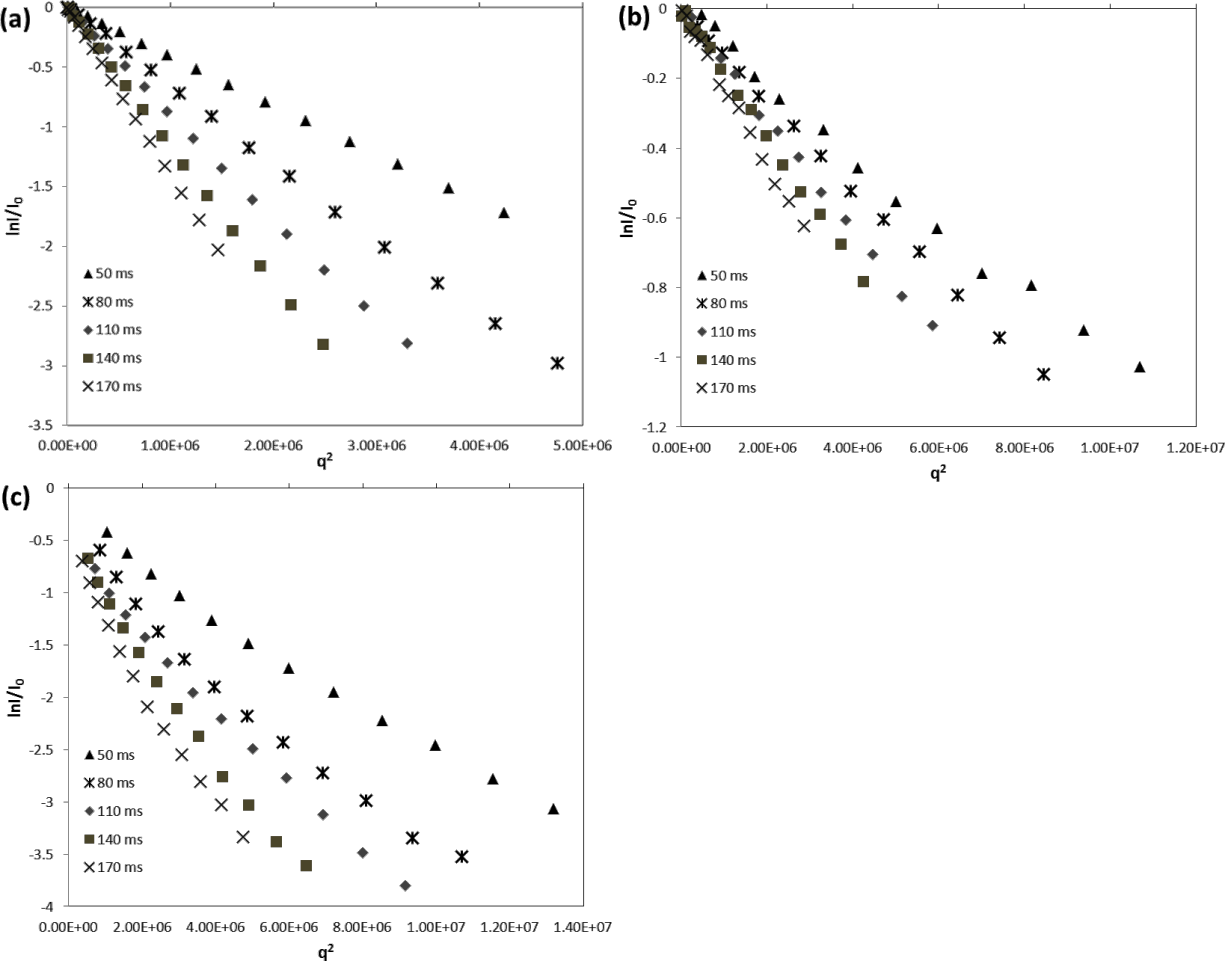
Normalized NMR signal decay *I(q,t*_d_*)* as function of *q^2^* for a) IP in D_2_O solution, b) IP in CDNSEDTA (1:4), c) IP in CDNSEDTA (1:8).

After having carried out a collection of experiments at increasing values of *t*_d_, the normalized experimental signal decays *I(q, t*_d_*)/I(0, t*_d_*)* can be plotted on a semilogarithmic scale as function of *q^2^* (Figure 2) for the set of diffusion times used, *t*_d_ = 50–170 ms in our case. The plots are reported in Figure 2. The liquid sample of IP dissolved in D_2_O (Figure 2a), as expected for a pure isotropic liquid solution, shows a linear dependence. The decay curves are obtained for the IP-CDNSEDTA (1:4) and IP-CDNSEDTA (1:8) are shown in panels b and c.

**Figure 3:**
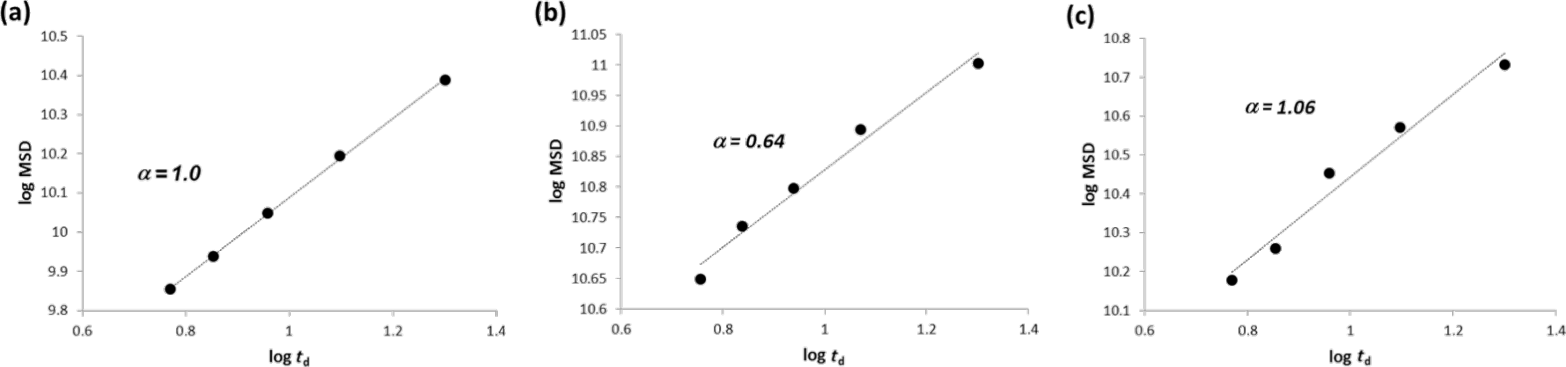
log–log plot of MSD vs diffusion time *t*_d_ for: a) D_2_O solution, b) CDNSEDTA (1:4) and CDNSEDTA (1:8).

From the data summarized in Figure 2 for the three samples studied, the MSD was calculated for several diffusion time *t*_d_ values as function of *q*^2^ (Equation 2). It is important to note that a log–log plot based on Equation 2 provides the experimental α values as the slope of the linear regression. In other words, the log–log plots provide immediate indication of the normal or anomalous (sub- or superdiffusive) dynamic regimes of our substrate confined in CDNS gels. The log–log plot is reported in Figure 3 for each case. A scaling exponent α = 1 is obtained for IP dissolved in D_2_O solution, thus indicating a Gaussian motion in the liquid solution. The IP drug in gel system CDNSEDTA (1:4) shows α = 0.64, indicating the presence of anomalous diffusion with clear subdiffusive characteristics. IP in CDNSEDTA (1:8) affords α = 1.06, thus indicating that also in this case IP experiences anomalous diffusion towards a superdiffusive regime. However, the small deviation of the observed α from 1 is a caveat for the over-interpretation of the data and suggests a thorough analysis of the MSD values.

The measured MSD are listed in Table 2 for five different diffusion delays Δ. In the first column the MDS data are related to the behaviour of IP in water solution with a non-anomalous diffusion regime. The ratio MSD (170 ms)/MSD (50 ms) can be interpreted as the increment of the average surface explored by IP in the time interval (120 ms). Such ratio for IP in water, undergoing non-anomalous diffusion, is 3.41. The analogous ratios for IP in CDNSEDTA (1:4) and CDNSEDTA (1:8) are 2.25 and 3.59, respectively. The former ratio is a clear indication of a deceleration effect with respect to the water solution, in good agreement with the subdiffusive regime previously mentioned on the basis of α = 0.64. The value obtained for CDNSEDTA (1:8) is greater than the reference found for IP in water and supports the hypothesis of anomalous diffusion with a slightly dominant superdiffusive effect.

**Table 2:** MSD (m^2^) of IP dissolved in: solution, CDNSEDTA (1:4) and CDNSEDTA (1:8) gel system at variable observation time *t*_d_ (s). Estimated experimental errors: 0.5% for the solution, 1.6% for CDNS 1:4 and 2.3% for CDNS 1:8.

Δ (s)	MSD solution (m^2^)	MSD CDNS 1:4 (m^2^)	MSD CDNS 1:8 (m^2^)

0.05	4.10E^−11^	9.93E^−12^	1.85E^−11^
0.08	6.38E^−11^	1.28E^−11^	2.69E^−11^
0.11	8.96E^−11^	1.59E^−11^	3.52E^−11^
0.14	1.15E^−10^	1.84E^−11^	5.51E^−11^
0.17	1.40E^−10^	2.25E^−11^	6.65E^−11^

After examining the results of Figure 3 and Table 2, some general and unprecedented conclusions can be drawn: i) the same observed molecule (IP) displays significantly different diffusive regimes in polymeric gels of cross-linked CDs. The transition from subdiffusive to (slightly) superdiffusive behaviour seems to be triggered, in the present study, by the polymer preparation protocol only, having kept other factors – concentration, temperature – constant. Additionally, as discussed in the next paragraphs, the pH values measured in the two systems span a limited range (6.5–6.9, vide ultra). ii) As a consequence, a modulation of the diffusivity of a given substrate in the polymer gels can be achieved by suitable polymer synthesis. This fact opens the possibility of a rational design of drug delivery/controlled release systems by controlling, inter alia, the transport properties of the encapsulated drug. iii) HRMAS NMR turned out to be a direct, efficient and quick method to gain diffusivity data on API loaded in complex systems resembling the formulations actually used for drug delivery, targeting or controlled release.

**Figure 4:**
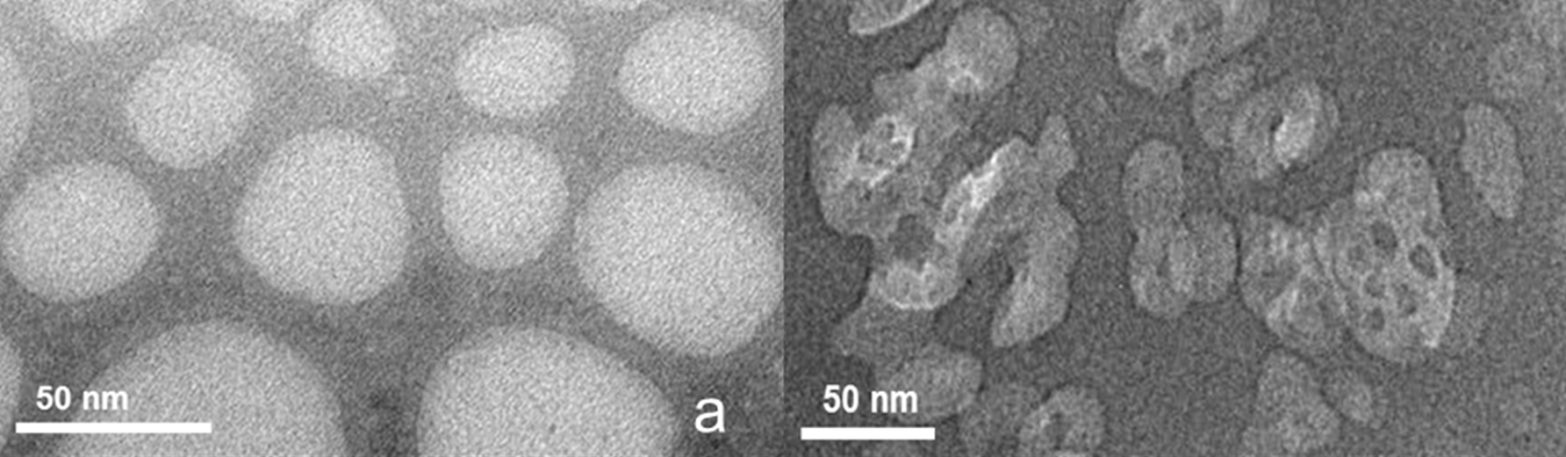
TEM images of: a) CDNSEDTA (1:4) and b) CDNSEDTA (1:8).

Finally, the important issue of how subdiffusive and superdiffusive regime can be related to the structure of the gel should be addressed. In principle, the subdiffusive behaviour can be related to the restricted diffusion of IP in the polymeric network due to the presence of nanopores originated during the cross-linking process. A visual description of the nanoporous structure of CDNSEDTA 1:4 is shown in Figure 4a.

The TEM image was recorded on samples stained with Pb(NO_3_)_2_ to achieve sufficient contrast. The picture clearly illustrates the porous morphology of the material and the structural heterogeneities in the pore size distribution. Confinement effects are expected to drive the diffusion process from normal to subdiffusive behaviour and interaction with the polymeric constituents.

**Figure 5:**
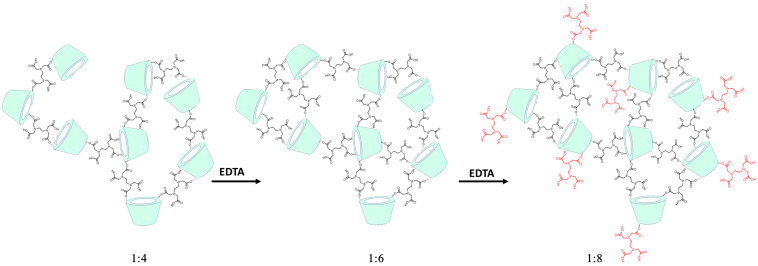
Effect of the increasing amount of crosslinker with respect to CD (expressed here as mol of crosslinker per mol of CD) on CDNS structure. The cross-linking degree increased up to 1:6, then further excess of EDTA causes branching of CD units rather that further cross-linking.

The interpretation of the superdiffusive regime observed for IP in the CDNSEDTA 1:8 is less straightforward. In order to account for the acceleration effect observed, we remind here that the cross-linking process in CDNS showed a marked dependence on the CD/cross-linker molar ratio. In a previous work published by our group [32], the FTIR and Raman bands in the 1680–1800 cm^−1^ wavenumber range were deconvoluted in the two sub-bands ω_CO1_ (centered at 1730 cm^−1^) and ω_CO2_ (centered at 1750 cm^−1^) assigned to the stretching mode of C=O in the ester and in the carboxylic acid functional groups, respectively. The associated intensities I_CO1_ and I_CO2_ were related to the populations of the two types of oscillators, namely COOR and COOH. The quantitative analysis of the intensity ratio I_CO1_/I_CO2_ showed that for molar excess less than a six-fold excess of EDTA with respect to CD (1:6) the cross-linking process was dominating. Larger excess of crosslinker (e.g., molar ratio greater that 1:6), caused branching of CD units rather than increasing the cross-linking degree. The overall result is an increasing number of dangling EDTA units with free COOH groups at the end in the CDNS prepared with a large excess of crosslinking agent. A simple scheme of these processes is shown in Figure 5.

The ionization state of the COOH groups is thus expected to play a key role in determining the possibility of a negative electrostatic potential in part of the polymeric backbone, in turn a possible cause of electrostatic acceleration leading to superdiffusive behaviour of the analyte IP. The ionization state of IP is also playing a pivotal role in such mechanism, due to the fact that a negative charge on IP may interact with the negative potential of the polymeric matrix.

The pH measured in the two gels loaded with IP – CDNSEDTA 1:4 and CDNSEDTA 1:8 – were 6.9 and 6.5, respectively. Taking into account the literature p*K*a values [33] of EDTA for the ionization of the four COOH groups ( p*K*_a1_ = 0.0, p*K*_a2_ = 1.5, p*K*_a3_ = 2.0, p*K*_a4_ = 2.7), the contribution of the two NH^+^ groups (p*K*_a5_ = 6.1, p*K*_a6_ = 10.4) and the p*K*a value of IP in its acid form (4.91), it is reasonable assuming that the majority of the COOH groups present in our systems are, in the pH interval of our preparations, present in the COO^−^ form and that one NH^+^ group is significantly contributing. As a consequence, an overall negative electric potential is expected in some parts of the network. Electrostatic acceleration effects on the random motion of the negatively charged IP molecules are taking place, thus providing the driving force for the superdiffusive component, as already found and reported in structurally related systems [34]. However, as previously underlined, the superdiffusive component is quite small. A simple electrostatic model seems to be too approximated to justify the experimental data. Rather, a complex superimposition of both superdiffusive and subdiffusive components seems a better description of the systems. The structure of the CDNSEDTA 1:8 is expected to show a high degree of heterogeneity in the distribution of pores, due to rearrangements caused by the steric hindrance. The TEM image of Figure 4b shows the details of the pores heterogeneity and the sponge-like morphology. The slightly superdiffusive behaviour observed on IP in CDNSEDTA 1:8 can be rationalized as a complex balance of the confinement effects and the electrostatic acceleration, with the latter dominating on the former.

As a final remark, we mention that the pH variations occurring during the preparative steps affording the drug-loaded hydrogels may, in principle, alter the structure of the polymeric network. We are currently investigating this aspect by small angle neutron scattering (SANS) on gels at variable and controlled pH. The data collected at the Heinz Maier-Leibnitz large scale facility (Munich, Germany, KWS-2 spectrometer) are being processed and will be submitted elsewhere.

## Conclusion

In conclusion, the present study demonstrated that HRMAS NMR spectroscopy is a powerful method for the direct observation of molecular species confined in polymeric hydrogels. The transport phenomena of IP in CDNSEDTA systems were studied in terms of MSD at different observation times, highlighting the transition between a subdiffusive to a superdiffusive regime modulated by the polymer structure in a small pH interval (6.5–6.9). These findings, and the methodology described for their assessment, can be exploited for the rational design of smart systems for drug delivery and controlled release.

## Experimental

### Nanosponges synthesis

**Scheme 1:**
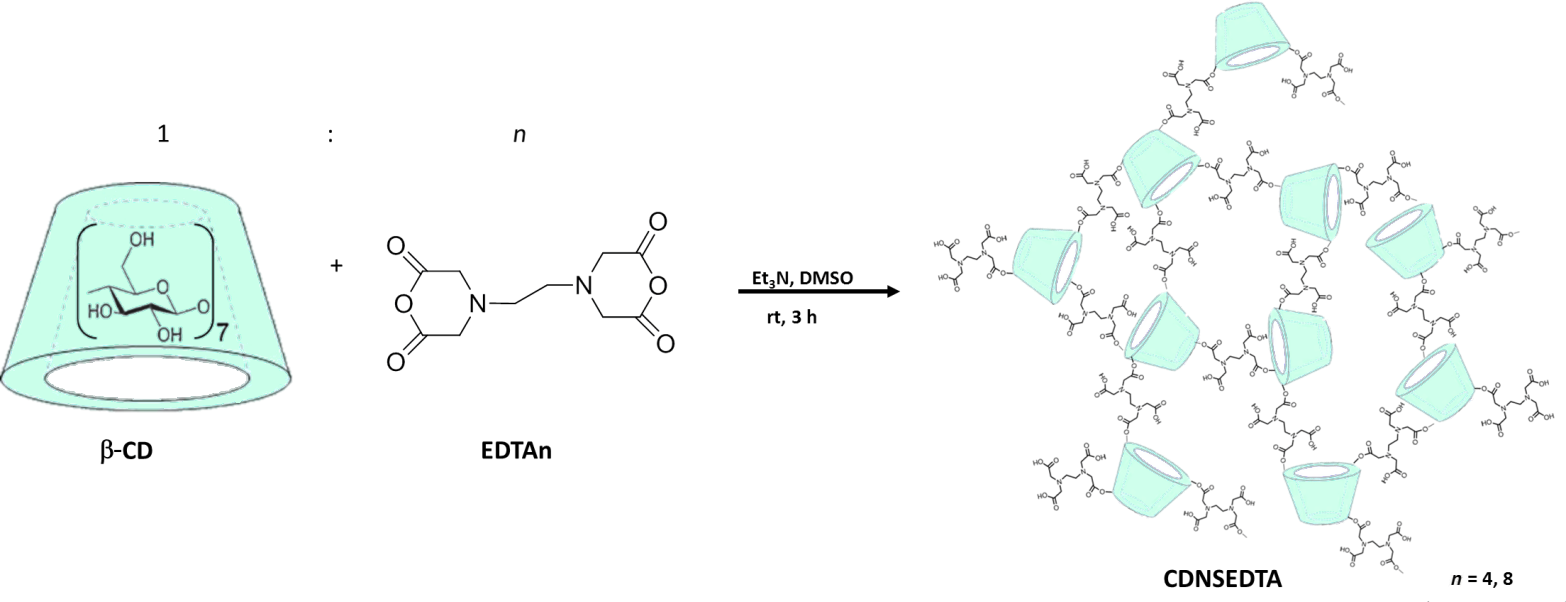
Schematic representation of the nanosponge synthesis. Acronyms: β-CD: β-cyclodextrin; EDTAn: anhydride of EDTA; CDNSEDTA: cyclodextrin nanosponge obtained by using EDTAn as cross-linker.

CDNSEDTA were prepared by reacting β-CD, dissolved in anhydrous DMSO and in the presence of anhydrous Et_3_N, with ethylenediaminetetraacetic acid dianhydride (EDTAn) at room temperature for 3 hours under intense stirring, as previously reported [17,31]. The obtained polymer was crushed in a mortar and purified by washings with 0.2 M HCl_aq_ (3 times) and deionized water (5 times) and finally dried under vacuum affording a homogeneous powder. The cross-linker EDTAn was added at molecular ratios 1:*n* (with *n* = 4, 8) giving two different products (Scheme 1).

### Drug loading and NMR samples preparation

The encapsulation of IP in CDNSEDTA 1:4 and CDNSEDTA 1:8 for the preparation of the loaded gels for HRMAS NMR analysis was done in three steps: (1) A 0.27 M stock solution of Ibuprofen sodium salt was prepared by dissolving IP in deuterated water (99.8%). An analogous water solution was prepared and its pH measured with a pH-meter giving the experimental pH value of 8.2. (2) The step (1) solution (150 μL) was added to a weighted amount (20 mg) of CDNSEDTA polymer in both preparations. (3) 2 mg of Na_2_CO_3_ (10% w/w) was then added to step (2) solution. After these steps, a perfectly homogeneous and transparent gel without any visible phase separation or solid particles was obtained in one hour. The preparations described above were repeated on a larger scale and by using H_2_O in order to obtain sufficient material for direct pH measurement with a pH-meter. The observed pH for CDNSEDTA 1:4 and CDNSEDTA 1:8 were 6.9 and 6.5, respectively. These values can be considered a good approximation of pD of the HRMAS NMR samples.

### HRMAS NMR spectroscopy

All the spectra were recorded on a Bruker Avance spectrometer operating at 500 MHz proton frequency, equipped with a dual ^1^H/^13^C HRMAS probe. Self diffusion coefficients were measured by diffusion ordered correlation spectroscopy (DOSY) experiments, based on the pulsed field gradient spin echo (PGSE) pulse sequence. The duration of the magnetic field pulse gradient (δ) in the z direction was optimized for each sample in order to obtain complete dephasing of the signals with the maximum gradient strength (G = 53.5 G cm^−1^). In each DOSY experiment, a series of 32 spectra with 32 k points were collected. For each experiment, 24 scans were acquired. Variable Δ measurements of the investigated samples were carried out by varying Δ in the range of 0.05–0.2 s, while the δ values were in the range of 1.4–3 ms. The pulse gradients were incremented from 2 to 95% of the maximum gradient strength in a linear ramp. The temperature was set at 305 K (32 °C). Data processing and fitting procedures were done by Dynamics Center software (2.1.8 version) (Bruker). The experimental error is between 2 and 3%.

### TEM

Images were recorded on a Transmission Electron Microscope Philips CM200 FEG at 200 kV electron accelerating potential. CDNSEDTA 1:4 and CDNSEDTA 1:8 were swollen with a solution of Pb(NO_3_)_2_ to obtain suitable gels containing the contrast agent and sampled on a 300-mesh copper grid coated with holey carbon film.
